# Impact of a High-protein Nutritional Intake on the Clinical Outcome of the Neurocritical Patients

**DOI:** 10.22037/ijpr.2019.112452.13766

**Published:** 2020

**Authors:** Forouzan Ahmadpour, Mehran Kouchak, Mir Mohammad Miri, Sara Salarian, Seyedpouzhia Shojaei, Kiana Ramezanzadeh, Paria Rezapour, Mohammad Sistanizad

**Affiliations:** a *Department of Clinical Pharmacy, Faculty of Pharmacy, Shahid Beheshti University of Medical Sciences, Tehran, Iran. *; b *Department of Critical Care Medicine, Imam Hossein Medical and Educational Center, Shahid Beheshti University of Medical Sciences, Tehran, Iran. *; c *Prevention of Cardiovascular Disease Research Center, Shahid Beheshti University of Medical Sciences, Tehran, Iran.*

**Keywords:** Neurologic disorders, High-protein diet, Prealbumin, Extended glasgow outcome scale, Nitrogen balance, APACHE-II score

## Abstract

Disease-related malnutrition of neurocritical illness harms its treatment, which increases the mortality rate. The aim of this study was evaluating the effect of a high protein diet on the dietary factors, clinical outcome, and mortality rate of neurocritical patients. In a randomized controlled trial, 15 neurocritical patients were recruited in each group. The patients in the intervention and control groups received high protein and conventional protein regimens, respectively. The Clinical Extended Glasgow Outcome Scale (GOSE) measured at one, two, and three months later. Acute Physiology and Chronic Health Evaluation II (APACHE-II) score, Glasgow Coma Scale, the serum level of transthyretin (TTR) on the first, 3^rd^ and fifth days of admission, and nitrogen balance (NB) at the baseline and fifth day of the study were recorded. Thirty patients, 15 in each group, entered into the study. There was no statistically significant difference in the baseline characteristics of the patients between the two groups of the study. The 28-days-mortality rate in the intervention and control group were 33.3% (n = 5) and 73.3% (n = 11), *P*-value = 0.034, respectively. The GOSE scores were higher in the patients who received a high protein diet, and lower in the patients with lower baseline TTR, higher APACHE-II score, older age, and a baseline negative nitrogen balance. The high protein diet may decrease the mortality rate, and improve the clinical outcome of neurocritical patients. The baseline TTR level, APACHE II score, and NB are prognostic factors for the prediction of the GOSE in neurocritical patients.

## Introduction

The prevalence of malnutrition, which results from changes in body composition, mechanical ventilation, insufficient nutritional input, an increased need, an altered utilization of nutrients, and disease-related malnutrition is estimated to be about 40% for the patients who admitted to the intensive care unit (ICU) (1-5). Malnourished patients suffer from inflammation, endocrine stresses, malfunctioning organs, pathological laboratory values, an impaired immune system, decreased body mass, undesirable clinical outcomes, and increased mortality rate. Disease-related malnutrition of neurocritical illness is one of the major causes of morbidity and mortality, and that increases the infection rates, prolongs the recovery time, and hospital stay (6-8). The annual mortality rate of undernourished hospitalized patients reported about 30% of cases (9). The primary purpose of nutritional therapy in neurocritical patients is to identify, prevent, treat malnutrition, and its complications. Therefore, they need special attention concerning nutritional care (10). There is little information about nutritional care in the neuro ICU patients. Hyper-metabolic status in neurological patients leads to a reduction in body fat and protein storage. Therefore, these patients need more protein and energy than other patients (11). Previous studies demonstrated patients with traumatic brain injury (TBI) needs about 100–140% of resting energy expenditure (REE) (12). Similarly, nontraumatic intracranial hemorrhage and spinal cord injury require more energy than the other patients (13, 14). Transthyretin (TTR) and Nitrogen balance (NB) are the best nutritional biomarkers. In a patient with calorie-protein malnutrition, the serum level of TTR decreases. But in a stressful situation, TTR has an indirect relationship with protein and calorie intake also, the NB falls and becomes negative (15). The result of the different studies showed the baseline TTR serum level, and NB is the predicting factor for the clinical outcome of critical patients (16-18). The use of high protein nutrition intake in the neurocritical patients associated with improved clinical outcomes (11). The effect of high protein intake on the clinical outcome of neurocritical patients is a new discussion on the nutritional field. So far, one clinical trial was done about it (19). This study investigated the effect of high protein intake on the clinical outcomes and mortality of neurocritical patients, and examined the relationship between nutritional biomarkers and their changes with the clinical outcome of patients.

## Experimental

This study was a prospective, randomized, open-label clinical trial in neurocritical patients admitted to the Imam Hossein Hospital, Shahid Beheshti University of Medical Sciences, Tehran, Iran, between September 2017 and February 2019. The ethical approval number and clinical trial number were IR.SBMU.PHNM.1396.879 and IRCT20120703010178N14, respectively. A total of 30 patients, fifteen in each arm, were studied. All of the patients entered the study after signing the written consent. The neurocritical patients (older than 16 years) with an estimated intensive care unit (ICU) stayed for more than five days and they were able to tolerate the enteral tube feeding included in the study. The exclusion criteria were death during the first five days, encephalopathy, pregnancy, metabolic disorders, acute and chronic kidney failure, and proteinuria. The patients randomly (by generating unique random numbers performed using the RAND function in Excel software) were divided into the intervention and control groups, who received high protein and conventional protein regimens defined as protein intake of 1.5-2 g/kg/day and 0.5-1.5 g/kg/day, respectively for five days by a nasogastric tube. To provide calories, the patients in both groups received standard enteral formula. In the intervention group, within 24-48 h after admission, protein intake has reached the goal, using V.M protein food supplement sachets (manufactured by Iran Daroo). The GCS is a neurological scale for assessing the state of a personal level of consciousness, and that is composed of three tests: eye, verbal, and motor responses with a range of points between three up to 15 (20). The APACHE II score is a severity-of-disease classification system with a range of points 0 to 70 and predicts a mortality rate of ICU patients. Higher scores indicated more severe disease with a higher risk of death. This score can be used to compare the outcome of different patients (21). The ICH Score composed of GCS, age, and initial neuroimaging (ICH volume, IVH, infratentorial/supratentorial origin and gives a score between zero to six which predicts mortality of the patients with ICH (22). The modified Fisher scale is a method for grading subarachnoid hemorrhage (SAH) based on a CT scan of the brain. The World Federation of Neurological Surgeons (WFNS) grading scale for subarachnoid hemorrhage (SAH) based on the GCS and focal motor deficit (23). The marshall score is a method for assessment of the TBI patients based on CT scans (24). Nitrogen balance is a measure of nitrogen input (protein intake/6.25) minus nitrogen output (urine nitrogen+4) (25). The GOSE is a scale for the functional outcome with eight categories: dead, vegetative state, lower severe disability, upper severe disability, lower moderate disability, upper moderate disability, lower good recovery or upper good recovery (11). 

 For all of the patients the data include age, sex, weight, body mass index (BMI), past medical, drug and habitual history, Acute Physiology and Chronic Health Evaluation II (APACHE II) score, daily calorie and protein intake, daily Glasgow Coma Scale (GCS) and baseline and fifth day nitrogen balance recorded. The ICH score, the modified Fisher scale and World Federation of Neurological Surgeons (WFNS) Subarachnoid Hemorrhage (SAH) Grading, and the Marshall score were used to evaluate the severity of the disease in the ICH, SAH, and TBI patients, respectively. The location and size of brain tumors were recorded. 

 Venous blood samples were collected from all of the patients to determine prealbumin (TTR) levels that were measured by enzyme-linked immunosorbent assay using a human TTR ELISA kit (Binding site, Diagnostics) according to the manufacturer instructions on 1^st^, 3^rd^ and 5^th^ day of admission. The Extended Glasgow Outcome Scale (GOSE) was evaluated by a call (one, two, and three months after the start of the study). 

 Data analysis was performed by the Statistical Package for the Social Sciences (SPSS, version 20.0; IBM Company). Data reported as means ± standard deviation (SD), and the *P*-value of < 0.05 was considered statistically significant. The Shapiro-Wilk test was used to assess data normality. The chi-square test, independent sample *t*-test, Generalized Estimating Equations (GEE) test employed for the determination of differences between the groups. Spearman correlation coefficient used for the analysis relationship between different parameters. 

## Results

**Figure 1:**
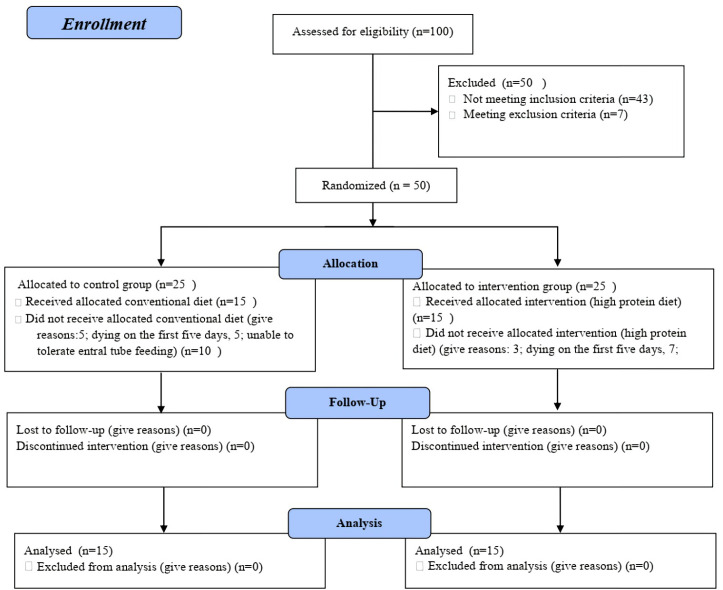
Disposition of patients throughout the study

The study protocol was presented in Figure 1. As shown in Table 1, there was no statistically significant difference in the baseline demographics and clinical characteristics including; GCS, nitrogen balance, TTR and APACHE II score, ICH score, the marshall score, SAH grading of the patients in two arms of the study.

**Figure 2:**
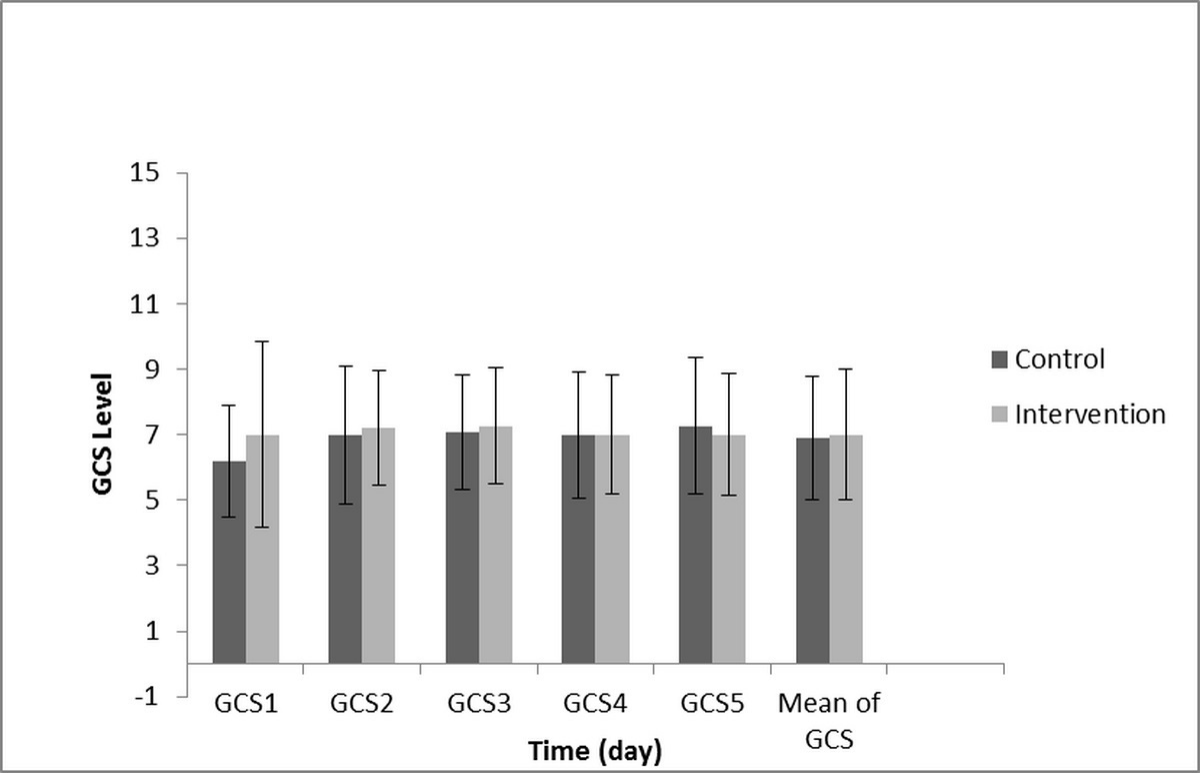
The comparison of the GCS score on different days between control and intervention group. These compared with the Tukey test *post-hoc*. There is no significant difference in the GCS level of patients between different days intergroup or intragroup

Because of the GCS, calorie, protein, TTR, and GOSE data did not have a normal distribution, and these were longitudinal/clustered data, for this reason the GEE model was selected for data analysis. The GEE test revealed no significant difference in the GCS between both groups in the different days of the study (*P*-value = 0.749). Also, GCS changes from the first to the fifth day showed no significant difference between both groups. (*P*-value = 0.702) (Figure 2).

**Figure 3:**
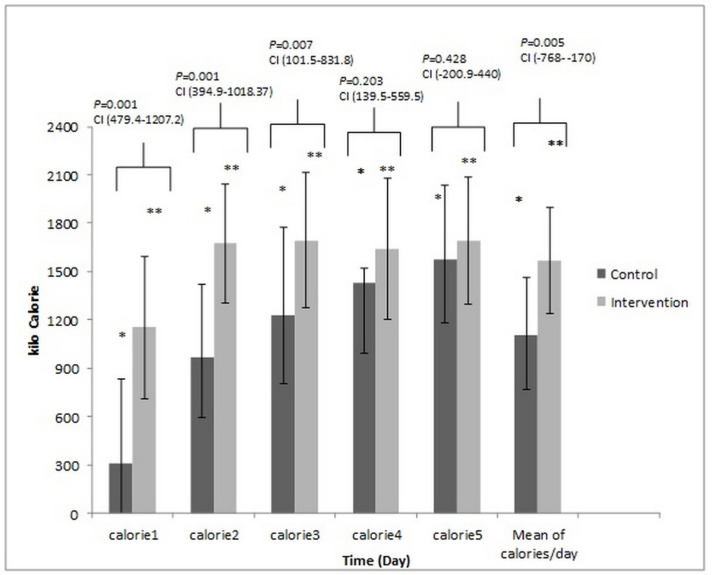
The comparison of the calorie received on different days between control and intervention group. These compared with the Tukey test *post-hoc*. The * Significant difference in the control group and the ** Significant difference in the intervention group between the Calorie/day received on the second, third, fourth and fifth day to the first. *P*-value = 0.001

**Figure 4:**
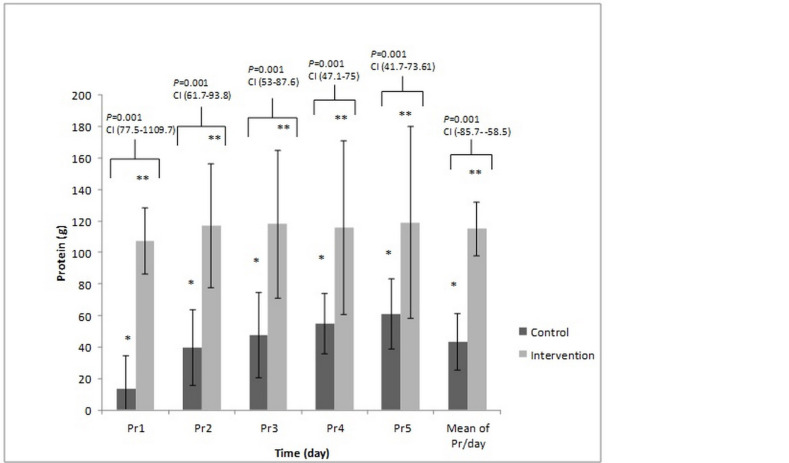
The comparison of the protein received on different days between control and intervention group. These compared with the Tukey test *post-hoc*. The * Significant difference in the control group and the ** Significant difference in the intervention group between the protein/day received on the second, third, fourth and fifth day to the first. *P*-value = 0.001

Figures 3 and 4 presents the total amount of energy and protein administered in two arms of the study. These compared with the Tukey test *post-hoc*. The mean calorie was 14.21 ± 5.65 kcalorie/kg/day *vs. *22.67 ± 5.23 kcalorie/kg/day; *P-*value = 0.001, and the mean protein intake was 0.54 ± 0.22 g/kg/day *vs.* 1.64 ± 0.14 g/kg/day, (*P-*value = 0.001) in the control and intervention groups, respectively.

Nitrogen balance (7.62 ± 5.22 *vs.* -2.33 ± 4.74 *P*-value = 0.001) in the fifth day and its change compared to baseline (18.10 ± 5.02 *vs.* 6.10 ± 5.22; *P*-value = 0.001) were significantly higher in the intervention group compared to the control arm of the study. These were analyzed with the *t*-test.

 The comparison of serum level of TTR (mg/dL) with the Tukey test *post-hoc* between two arms of the study, did not reveal any significant difference in the third (135.8 ± 104.0, 159.2 ± 131.0, *P*-value = 0.905) and the fifth (108.6 ± 85.0, 146.4 ± 70.0, *P*-value = 0.709) day of the study between control, and intervention arm, respectively. Also, the change of the TTR from the first to the fifth day of the study did not reach a significant level (22.0 ± 148.0 *vs.* 52.0 ± 69.0, *P*-value = 0.686 in the control and intervention groups, respectively). 

**Figure 5:**
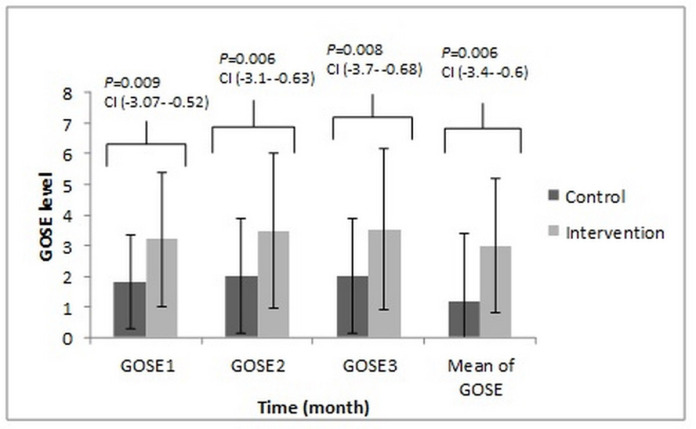
The comparison of GOSE on different months between the control and intervention groups. These compared with the Tukey test *post-hoc*

 In 28 days follow up, 11 (73.3%) and five (33.33%) of the patients expired in the control and intervention groups, respectively (*P*-value = 0.037). This data was compared with the chi-square test. The mean ICU length of stay (LOS) of control and intervention arm was 27 ± 3 and 25 ± 5, respectively (*P*-value = 0.112, this data was compared with the *t*-test.). The Tukey test *post-hoc* revealed a significant difference in the GOSE outcome between two groups in the different months of the study (Figure 5). The results of the Spearman correlation analysis indicated a significant association between a high APACHE-II score and the low baseline TTR level for all of the patients (r = -0.443, *P*-value = 0.047). In the control group, analysis indicated a significant association between the low baseline TTR level and worsened the GOSE outcome for three months follow up (r = 0.585, *P-*value = 0.022) while in the intervention arm analysis do not indicate a significant relationship but, the results indicated a significant association between positive changes of the TTR level from the first to the fifth day with positive changes of the GOSE from the first to the third month (r = 0.562, *P-*value = 0.029). The results of the control arm indicated negative changes in the TTR level from the first to the fifth day without positive changes in the GOSE score. Just, in the intervention group the positive change of the TTR level during five days had a significant association with the mean of calorie/day and protein/kg/day, respectively (r = 0.640, *P-*value = 0.045; r = 0.549, *P-*value = 0.034), and just in the intervention group the results of analysis showed that the positive changes in the GCS during five days had a significant association with the mean of the calorie/day (r = 0.548, *P*-value = 0.034). In both groups, the nitrogen balance in the fifth day had a significant association with the mean of calorie/kg/day and protein/kg/day, respectively in the intervention group (r = 0.783, *P*-value = 0.001; r = 0.789, *P*-value = 0.001) and in the control group (r = 0.545, *P*-value = 0.030; r = 0.698, *P*-value = 0.004). The results also, indicated negative correlations between APACHE II score and baseline nitrogen balance (r = -0.540, *P*-value = 0.039 and r = 0.563, *P*-value = 0.029 in the intervention and control groups), baseline TTR (r = -0.543, *P*-value = 0.009 and r = -0.585, *P*-value = 0.022 in the intervention and control arms of the study) and the GOSE outcome on the 3^rd^ month of the study (r = -0.630, *P*-value = 0.012 and r = -0.585, *P*-value = 0.022 in the intervention and control groups, respectively). Also, in the outcome assessment the results showed an older age (The intervention group; r = -0.566, *P-*value = 0.023 and the control group r = -0.728, *P*-value = 0.002), a higher APACHEII score (The intervention group; r = -0.631, *P*-value = 0.012 and the control group r = -0.512, *P*-value = 0.044), a lower baseline TTR level (The control group r = 0.582, *P*-value = 0.022) and a baseline negative nitrogen balance (The intervention group; r = 0.654, *P*-value = 0.009 and the control group r = 0.543, *P*-value = 0.022) and the longer ICU length stay (The intervention group; r = -0.630, *P*-value = 0.012, the control group r = -0.557, *P*-value = 0.031) had a significant association with worsen clinical GOSE outcome.

## Discussion

 Although several trials and meta-analyses have previously examined the effect of a high protein diet in ICU patients (19). This study is the second RCT that examined the effect of a high protein diet in neurocritical patients. In this randomized clinical trial, we evaluate the impact of a high protein diet on the nutritional biomarker (nitrogen balance and serum level of TTR) and clinical status in neurocritical patients. The sample groups matched concerning demographic and clinical characteristics such as age, sex, BMI, APACHE II score and baseline of GCS, ICH score, SAH grading, and the Marshall score of TBI patients. Our findings revealed that the 28-day mortality rate was significantly lower in the intervention group. We observed a significant relationship between the higher APACHE II score, lower the baseline serum level of TTR, a negative baseline nitrogen balance, and older age with the lower GOSE outcome. Therefore, we came to that conclusion: a high protein regime may have a significant role in improving the GOSE outcome, and decreasing mortality rate because of a significant difference was observed in the nitrogen balance on the fifth day and three months GOSE clinical outcomes between both groups, and the baseline serum level of TTR, NB, age, and APACHE-II score are the prognostic factors for predicting GOSE outcome. APACHE II score is one of the most specific prognostic factors for ICU patient. This standard score is useful for predicting the mortality rate in the ICU patients with a different diagnosis (21). Also, we used this score to compare the severity of the disease and mortality rate in our patients with a different mechanism of brain injury. Since, due to the nonsignificant difference of this score between the two groups, we can ignore the variation in the mechanism of brain injury that was considered as a confounding factor. In a recent study, the results showed the performance of the APACHE-II score was higher discrimination than the ICH score (22). Currently, available evidence on the good functioning of APACHE-II in the neurocritical patient is available (26, 27). The initiate GCS has become a fundamental aspect of the clinical care of the patients with TBI (GCS), aneurysmal SAH (HuntHess and WFNS), ICH patients (ICH score) and ischemic stroke (NIHSS) (22). Therefore, in our study, the initiate GCS of the patients was recorded and used for standardized assessment of all of the patients. Also, to further evaluate the severity of the disease in each patient category; ICH score, SAH grading, the Marshall score of TBI patients, size and location of tumors recorded that there was no difference between the two groups. Malnutrition can cause multi-organ failure, more infection, and prolonged mechanical ventilation dependency. A higher protein diet can reduce these complications and a better clinical outcome (19). Similarly, the 28-day mortality rate is significantly lower in the intervention group in our study. Similar to our findings, previous studies indicated age, and the APACHE-II score is the prognostic factors about the clinical outcome (28). In a clinical trial 118 ICU patients were examined, the result showed that the serum level of TTR on the third and seventh days of ICU admission did not increase, although the patients received enough calories and protein intake (29). Similar to the results of our study, the serum levels of TTR did not have a significant difference on different days in two groups and intragroup. In a clinical trial, the results showed that the patients with the lower baseline serum TTR before surgery had the longest stay in the hospital and ICU. Also, they had higher mortality and infection rates (15). Until 2018, only two studies examined the effect of a high protein diet on the clinical outcome in neurocritical patients, and only four clinical trials examined the effect of a high protein diet on the medical or surgical adult ICU patients (19). In the first study, Clifton *et al*., 20 head trauma patients were studied in two different groups. But there was no difference in the mortality rate and clinical outcome. But the results of our study were contrary to this study. This may be due to the following reasons; in the Clifton study, the control group received 14%, and the intervention group, 22% of its calories as protein but in our study protein was not used to provide calories. Also, in the Clifton study, the mean of nitrogen balance during seven days of the study was negative in both groups, but in our study, the mean of nitrogen balance of intervention group became positive (30). In the Second study, Oertel *et al.* reported that the GOS scores were higher when neuro ICU patients received a protein-rich diet (11). Also, the result of the different studies showed the TTR serum level was the predicting factor for the clinical outcome, and mortality after surgery, in TBI and stroke patient. In a clinical trial, 117 ischemic stroke patients were examined, and the results showed that the patients who had lower serum levels of TTR, had a lower GOSE outcome (31). In another study, 81 ischemic stroke patients were examined, and the results showed the baseline serum level of TTR was an important predicting factor for the one-year mortality and clinical outcome (32). The similar results were reported in different studies. Also, in different studies, the results showed the baseline serum level of TTR had a direct relationship with an Injury Severity Score (ISS), and an APACHE-II score and higher ISS or APACHE-II is associated with lower TTR levels. Similar results were obtained from our study (16-18). Jivnani *et al.* reported the hypercatabolic state after brain injury can cause a negative nitrogen balance and rapidly start of central feeding can cause an increase in nitrogen balance and better clinical outcome (17). Nataloni *et al.* also, reported a direct relationship between nitrogen balance and the severity of the injury. Other studies demonstrated that a negative nitrogen balance had a significant association with the worsened clinical outcome (18). A similar result was found in our study. In this randomized clinical trial, we evaluated the effects of a high protein diet on the nutritional biomarkers and clinical status in neurocritical patients, and a significant difference was observed in the nitrogen balance on the fifth day, three-month GOSE clinical outcome and mortality rate between both groups. Unfortunately, the different prognosis of various diseases is a confounding factor in our study and can affect the significance of the result. Although the APACHE II score is intended to evaluate the uniformity of severity of injury between the two groups, and there was no significant difference between the two groups. Also, the severity of the disease in each patient category; ICH score, SAH grading, the Marshall score of TBI patients, size and location of tumors evaluated, and there was no significant difference between the two groups. Because of the limited sample size that was related to the single-center study, and a long period to complete this sample size, we believe that the findings provide an additional benchmark for further studies involving more patients and longer duration of intervention.

**Table 1:** Demographic and clinical data of patients in intervention and control groups

**Group**							
**Parameter**				**Intervention**	**Control**		***P-*** **value**
Mean age (years)				47 ± 18	53 ± 21		0.460^**^
Sex (male), n				7 (46%)	7 (46%)		0.999^*^
BMI, kg/m^2^				28.0 ± 14.0	28.0 ± 5.5		0.137^**^
History of abuse, n				3 (20%)	4 (26%)		0.523^*^
Drug history, n	Anti-hypertensive			6	3		0.712^*^
	Anti-platelet			4	4		
	Anti-diabetic			4	3		
	Levothyroxine			1	1		
	Anticonvulsant			0	2		
Past medical history, n	Depression			0	1		0.710^*^
	Diabetes			3	5		
	Heart disease			11	10		
	Seizure			1	0		
	Hypothyroid			1	1		
Admission diagnosis n (%)	ICH-IVH		n (%)	6 (40%)	9 (60%)		0.179^*^
			ICH-score	2.6 ± .5	2.5 ± .5	*P* = 0.789^**^	
	TBI		n (%)	4 (26/6%)	4 (26/6%)		
			The Marshall score	2.3 ± .3	2.6 ± .4	*P* = 0.456^**^	
	BT^a^			4 (26/6%)	1 (6.6%)		
	SAH	N (%)		1 (6/6%)	1 (6/6%)		
		The modified Fisher scale		Grade III	Grade III		
		WFNS		Grade V	Grade IV		
APACHE II score, (range)				24.00 ± 4.46 (18-32)	23.00 ± 5.71 (16-34)		0.590^**^
The baseline GCS score (range)				6.20 ± 1.70,	7.00 ± 2.85		0.334^***^
The baseline NB				-8.43 ± 5.80	-10.00 ± 2.52		0.215^**^
The baseline TTR				116.13 ± 53.00	124.13 ± 134.00		0.802^**^

Data showed as the mean ± SD and n (%). ^*^P-value calculated with the chi‑square test; ^**^P-value calculated by *t*-test. ^***^P-value calculated by GEE. ^a^low-grade glioma in the parietal and frontal lobe. N: number. SD: standard deviation; BMI: body mass index; IVH: intraventricular hemorrhage; ICH: intracranial hemorrhage; TBI: traumatic brain injury; BT: brain tumor; CVA: cerebrovascular accident; SAH: subarachnoid hemorrhage; APACHE II: acute physiology and chronic health evaluation II; GCS: glasgow coma scale; NB: nitrogen balance; TTR: transthyretin.

## Conclusion

 High protein diet in neurocritical patients may reduce mortality and associate with the better GOSE clinical outcome, while the higher calorie intake has a positive effect on the TTR level and a GCS score during ICU stay. The APACHE II score, age, the baseline serum TTR level, and the baseline nitrogen balance in these patients, all are the prognostic factors in the predictions of mortality and GOSE clinical outcome. The limitation of our study was a low sample size; then we suggested a larger study in the future.
